# Integrated genetic and epigenetic analysis reveals DNA repair alterations in multifocal hepatocellular carcinoma

**DOI:** 10.1038/s41392-023-01446-z

**Published:** 2023-06-23

**Authors:** Yi-Hong Ling, Meng-Ni Liu, Yi-Xin Yin, Zhong-Guo Zhou, Jie-Wei Chen, Wei Wei, Jing-Ping Yun, Dan Xie, Rong-Ping Guo, Mu-Yan Cai

**Affiliations:** 1 State Key Laboratory of Oncology in South China, Collaborative Innovation Center for Cancer Medicine, Sun Yat-sen University Cancer Center, Guangzhou, China; 2 Clinical Big Data Research Center, Scientific Research Center, The Seventh Affiliated Hospital of Sun Yat-sen University, Shenzhen, China

**Keywords:** Cancer genomics, Tumour heterogeneity

**Dear Editor**,

Hepatocellular carcinoma (HCC) ranks the fourth most lethal cancer worldwide and over 50% of cases are diagnosed as multifocal HCC (mHCC) with dismal prognosis.^1^ mHCC displays more complicated intratumor heterogeneity (ITH) and clonal evolution course which decreases the efficacy of clinical treatments.^2^ DNA damage repair (DDR) alterations have been put forward to be a prominent contributor to ITH.^3^ However, little is known about the role of DDR alterations in the genetic and epigenetic evolution of mHCC.

To decipher the genetic ITH in mHCC, we performed whole-exome sequencing (WES) on multiple lesions (MLs) including primary tumor, satellite nodule and portal vein tumor thrombus of seven mHCC patients (Supplementary Fig. 1 and Supplementary Table 1). Overall, we found that both the mutational profile (Fig. 1a, b) and copy number alterations (CNAs) profile (Fig. 1c, d) were heterogeneous between lesions and between patients, indicative of substantive genetic ITH of mHCCs. *TP53*, *AXIN1, KEAP1*, and *TTN* are the most frequently mutated genes among the patients. The phylogenetic trees constructed by non-silent mutations of all patients showed branched evolutionary patterns (Fig. 1e). Compared to other patients, the primary tumors and satellite nodules of HCC3 and HCC4 exhibited more diverse mutation (Fig. 1a, b and Supplementary Fig. 2) and CNA patterns (Fig. 1c, d), and greater genetic distances in the phylogenetic trees (Fig. 1e). HBV DNA integration has been considered as the main contributor to HCC tumorigenesis. We further identified the HBV integration sites in HCC1, HCC3, HCC5, HCC6 and HCC7 (Fig. 1f and Supplementary Table 2). Among them, the primary and secondary lesions of HCC1, HCC5 and HCC6 shared common HBV integration sites, indicating that secondary lesions inherited the HBV integrations from their primary tumors. These genetic findings suggested that HCC3 and HCC4 follow multicentric origin (MO), while other patients are more likely to be intrahepatic metastasis (IM) (Supplementary Table 3). In line with the clinical manifestation, MO HCC patients seemingly have a favorable outcome compared to IM patients (Supplementary Table 1).

**Fig. 1:**
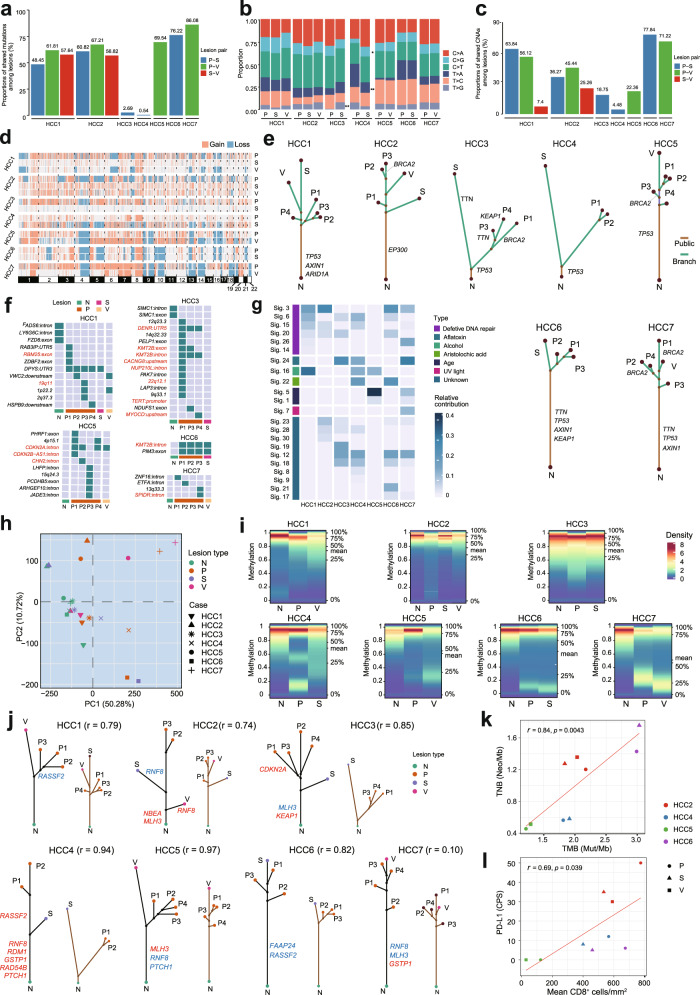
The comprehensive assessment of the genetic and epigenetic heterogeneity identified DNA damage repair (DDR) alterations as a ubiquitous feature in multifocal hepatocellular carcinomas (mHCCs). **a**, **b** Heterogeneous mutational patterns of seven mHCCs. Bar plots showing proportions of mutations shared by any pair of lesions in each patient (**a**). Stacked bar plots showing the mutation proportions of each lesion accounted for by each of the six mutation types. For each patient, a Fisher exact test was applied to evaluate the difference in mutation spectra between each pair of lesions. Significant *P* values are denoted by asterisks (**P  < 0.01, *P < 0.05) (**b**). **c**, **d** Heterogeneous CNA patterns of seven mHCCs. CNA profiles of all tumor lesions from seven mHCC patients. Each track represents one tumor lesion. CNA regions are colored either in red (gain) or blue (loss) (**c**). Bar plots showing proportions of CNAs shared by any pair of lesions in each patient (**d**). **e** Phylogenetic trees of seven mHCC patients. For each patient, the phylogenetic tree was constructed from all somatic nonsynonymous mutations using the NJ algorithm. Truncal mutations and branch mutations are represented with brown and green lines, respectively. The branch lengths are proportional to the number of mutations. Putative HCC driver genes and DDR genes are indicated along with trees. **f** The detection of HBV integration sites based on WES.Grid indicates the presence (green) or absence (gray) of HBV integration sites detected. The HBV integration sites verified by VISDB database are indicated in red. **g** The relative contribution of each COSMIC v2 signature for mHCC patient. **h**, **i** Heterogeneous epigenetic features of seven mHCCs. Principal component analysis of the methylation profiles based on the averagemethylation values in 5 kb titling regions of all normal tissue and tumor lesions from seven mHCC patients (**h**). Methylation density plots of seven mHCC patients (**i**). For each column that represents one sample in the plot, colors were mapped to the density values in the corresponding distribution. The black dashed lines correspond to the five quantiles of the distributions (0%, 25%, 50%, 75%, and 100%) and the red dashed lines correspondto the mean value of the distributions. **j** Phyloepigenetic trees of seven mHCC patients. For each patient, the phyloepigenetic tree was constructed based on the NJ approach using CpGs with at least 20 reads. For direct comparison, phylogenetic trees from panel **e** were reproduced next to the matched phyloepigenetic trees. Pearson’s correlation coefficient was employed to calculate the similarity between the genetic and epigenetic distance matrices for each patient and it is indicated on the plot. **k** Pearson’s correlation analysis was performed between TMB and TNB for HCC2, HCC4, HCC5 andHCC6. **l** Pearson’s correlation analysis was performed between CD8+T cell densities and expressions of PD-L1 (CPS score) for HCC2, HCC4, HCC5 and HCC6

To explore which mutational processes operate in the genetic evolution of mHCC, we further performed mutational signatures analysis. The overall mutational spectra were heterogeneous between lesions (Supplementary Fig. 3) and between patients (Fig. 1g). Interestingly, Signature 5 was observed in the lesions of the tumor thrombi (Supplementary Fig. 3). Notably, all cases except HCC5 had DDR-associated signatures including Signature 3, 6, 14, 15, 20, and 26, indicating that DDR alterations may play important roles in the evolution spectrum of mHCC (Fig. 1g). Mutations of DDR genes such as *TP53*, and *BRCA2* were frequently detected on the trunks and branches of the mHCC patients (Fig. 1e), suggesting DDR deficiency might be a ubiquitous feature in different stages of tumorigenesis. Of note, the DNA damage checkpoint gene, *TP53*, was mutated in all patients except HCC2, predominantly laid on the trunks of six patients. Specifically, *TP53* mutations were found to be clonal events in all lesions of four patients (HCC4, HCC5, HCC6 and HCC7) (Supplementary Table 4), highlighting the potential role of this gene as a founder in evolution. *BRCA2* mutations were identified as branched events in four patients, indicating that it might function in late evolution of mHCC. In the diverse functional categories and pathways of the mutated DDR genes, we found that *P53* pathway, nucleotide and excision repair pathways were frequently altered in the genetic evolution of mHCC (Supplementary Fig. 4). Taken together, our data have suggested that the genetic DDR alterations might fuel different stages of tumor evolution, which might provide novel insights into understanding the potential mechanism of ML evolution.

In addition to genetic alteration, epigenetic alteration, especially DNA methylation, has been demonstrated to play a significant role in cancer evolution.^4^ To dissect the epigenetic ITH of mHCC, we profiled the DNA methylation landscape of MLs using whole-genome bisulfite sequencing (WGBS). In line with genetic ITH, mHCC also exhibited a high level of methylation heterogeneity between lesions and between patients (Fig. 1h–i). Global hypomethylation was observed in mHCC lesions when compared to the paired normal liver tissue (Fig. 1i). Interestingly, the global methylation levels of tumor thrombi in HCC1, HCC5 and HCC7 were lower than those of the paired primary tumors (Fig. 1i). DNA methylation aberrations at CpG island (CGI)-promoters of driver genes have been reported to participate in reprogramming gene expression, contributing to tumorigenesis. In this study, we found that tumor suppressors (*PTCH1, RASSF2*, and *GSTP1*) and oncogenes (*KEAP1*) were variously methylated at their CGI-promoters (Fig. 1j). Interestingly, *KEAP1* was both mutated and methylated at the CGI-promoters, suggesting that *KEAP1* was disrupted during both the genetic and epigenetic evolutionary processes. To further dissect the epigenetic evolution of mHCC, we constructed the phyloepigenetic trees for each patient. Distinct lesions of all patients laid on different branches within the corresponding trees (Fig. 1j), indicating that genome-wide methylation heterogeneity is a ubiquitous feature in tumor differentiation. To decipher the potential relationship between the epigenetic evolution and genetic evolution of mHCCs, we then depicted the spatial distribution of epigenetic and genetic alterations in MLs and mapped their trajectory paths in tumor phylogeny. Interestingly, the topology between the phyloepigenetic trees and the corresponding phylogenetic trees of all patients were highly identical (Pearson’s correlation coefficient, range 0.74–0.97) except HCC7 (Fig. 1j), which underlined the interaction of genetic and epigenetic alterations during mHCC evolution and the co-dependency of these disparate machineries in tumorigenesis.

We next analyzed the alterations of DDR genes at the epigenetic level. The aberrant methylation of DDR genes including *RNF8*, and *MLH3* were detected on both the trunks and branches of the phyloepigenetic trees, predominantly on the trunks (Fig. 1j), suggesting that aberrant DNA methylation of these genes might take a significant part in early tumor evolution of mHCC. Collectively, our observation provided a great understanding of the importance of DDR alterations to the evolution of mHCC at the epigenetic level.

DDR alterations have been considered as important determinants of response to immunotherapy in cancers.^5^ Given that our findings were indicative of the importance of DDR gene alterations in the genetic and epigenetic evolution of mHCC, we further explored the relationship between DDR-associated signatures and immunotherapy response. Among four patients receiving anti-PD-1 immunotherapy, three patients (HCC2, HCC4, and HCC6) achieved partial response (PR) or stable disease (SD) while the other one (HCC5) achieved progressive disease (PD) (Supplementary Table 1). Notably, these three responders, including PR and SD patients, were characterized by DDR-associated signatures (Fig. 1g), and also exhibited higher tumor mutational burden (TMB) and tumor neoantigen burden (TNB) (Fig. 1k). To further profile the tumor microenvironment (TME) of mHCC, we assessed lymphocyte infiltration, PD-1 and PD-L1 expression using immunohistochemistry (Fig. 1l and Supplementary Fig. 5–6). We observed that the infiltrative CD8^+^T lymphocyte densities and PD-L1 expression were significantly higher in responders (PR and SD), compared to non-responder (PD) (Fig. 1l). Our data suggest that DDR alterations might result in increased TMB and TNB, and higher levels of tumor-infiltrating CD8^+^T cells, eventually contributing to tumor antigenicity and immunotherapeutic response.

In summary, the present work provided a comprehensive evaluation of the genetic and epigenetic ITH of mHCC and a novel understanding of the importance of DDR alterations that might be implicated in tumor evolution. Of the patients receiving immunotherapy, DDR alterations in mHCCs may be a potential predictor for immunotherapeutic efficacy.

## Supplementary information

Supplementary Materials

Data Set 1

## Data Availability

The datasets used and/or analyzed during the current study are available from the corresponding author upon reasonable request.
