# Supplementary material for: Integrated genetic and epigenetic analysis reveals DNA repair alterations in multifocal hepatocellular carcinoma
Source: Signal Transduct Target Ther. 2023 Jun 23;8:244. doi: 10.1038/s41392-023-01446-z (PMC10287708; doi:10.1038/s41392-023-01446-z)
Supplement: Supplementary file 1 — Supplementary Materials [file 41392_2023_1446_MOESM1_ESM.docx]

Supplementary Materials for

Integrated Genetic and Epigenetic Analysis Reveals DNA Repair Alterations in Multifocal Hepatocellular Carcinoma

Yi-Hong Ling**^#^**, Meng-Ni Liu**^#^**, Yi-Xin Yin**^#^**, Zhong-Guo Zhou**^#^**, Jie-Wei Chen, Wei Wei, Jing-Ping Yun, Dan Xie*, Rong-ping Guo*, and Mu-Yan Cai*

Correspondence to: [xiedan@sysucc.org.cn](mailto:xiedan@sysucc.org.cn); [guorp@sysucc.org.cn](mailto:guorp@sysucc.org.cn); caimy@sysucc.org.cn

**This PDF file includes:**

Materials and Methods

Discussion

Figure. S1 to S6

Table. S1 to S4

Materials and Methods

**Sample acquisition**

Thirty-three MLs (including 24 lesions of the tumor, five lesions of satellite nodule and four lesions of tumor thrombi in the portal vein) and seven matched adjacent non-tumor liver tissues were collected from seven mHCC patients. These patients underwent resection before any adjuvant therapy at Sun Yat-sen University Cancer Center between August of 2014 and April of 2016. Detailed clinicopathological parameters of seven mHCC patients were provided in Supplementary Table 1. All of the tissues were snap-frozen in liquid nitrogen immediately within 15 minutes after resection and preserved at -80 °C before sequencing analysis. Two pathologists (Yi-Hong Ling and Mu-Yan Cai) independently confirmed the diagnosis based on the World Health Organization (WHO) classification of Tumors of the Digestive System (2019 version).

**Whole exome sequencing**

The library was constructed using TaKaRa Kit according to the manufacturer’s protocol. Exome regions were captured with SureSelectXT Human All Exon V6 (Agilent Technologies) according to the manufacturer’s protocol. The post-hybridization amplification product (2×150-bp paired-end reads) was quality checked and sequenced with Illumina HiSeq 6000.

**Whole Genome Bisulfite Sequencing**

A total amount of 5.2 microgram genomic DNA spiked with 26 ng lambda DNA was fragmented by sonication to 200-300bp with Covaris S220, followed by end repair and adenylation. Cytosine-methylated barcodes were ligated to sonicated DNA as per the manufacturer’s instructions. Then these DNA fragments were treated twice with bisulfite using EZ DNA Methylation-GoldTM Kit (Zymo Research) before the resulting single-strand DNA fragments were PCR amplificated using KAPA HiFi HotStart Uracil + ReadyMix (2X). Library concentration was quantified by Qubit® 2.0 Flurometer (Life Technologies, CA, USA) and quantitative PCR, and the insert size was assayed on Agilent Bioanalyzer 2100 system. The library preparations were sequenced on an Hiseq X-Ten or Novaseq platform and 125bp/150bp paired-end reads were generated. Image analysis and base calling were performed with Illumina CASAVA pipeline, and finally, 125bp/150bp paired-end reads were generated.

**Exome sequencing data analysis for SNVs and INDELs calling**

Paired-end clean reads were aligned to the reference genome (UCSC hg19) using BWA-MEM (version 0.7.10) software with default parameters. Then we sorted aligned reads and marked for duplicates with Picard. Indel realignment and base quality recalibration were performed using Genome Analysis Toolkit (GATK version 3.3). In the next step, we conducted variant calling, filtering and copy number detection. Paired-end clean reads were aligned to the human reference genome (NCBI build hg19) using BWA-MEM v 0.7.10 software with default parameters^1^. Then we sorted aligned reads and marked for duplicates with Picard Tools v. 1.129. INDEL realignment and base quality recalibration were performed using Genome Analysis Toolkit (GATK v3.3.0). In the next step, we conducted somatic SNVs calling for each tumor/normal pair using Mutect v1.1.7^2^ in high-confidence mode. Detected variants with total depth ≤ 10 or variant reads in tumors ≤ 3 were discarded. We used Strelka v2.7.1^3^ together with Manta v 1.1.1^4^ for accurate INDELs detection. Those potential false positive calls of INDELs were filtered based on its empirical variant scoring (EVS) model (removing “LowEVS” calls). Both sSNVs and INDELs were annotated with ANNOVAR v.20150617^5^. The following filters were further applied to identify the SNVs and indels: (i) Mutations with <10 total reads or 3 variant reads were discarded. (ii) Mutations listed in dbSNP147 were removed unless they were documented in the Catalogue of Somatic Mutations in Cancer (COSMIC) database. (iii) Mutations listed in the National Heart, Lung, and Blood Institute Exome Sequencing Project were removed.

**Mutational signature analysis**

Base substitutions could be divided into 6 categories, namely, C > T, C > A, C > G, T > C, T > G, and T > A. Considering the 5′and 3′ flanking nucleotides of a specific mutated base, a total of 96 substitution types exist. For each patient, we compared the mutations within specific contexts between each pair of lesions using fisher exact tests. To extract the underlying mutational signatures, we then applied the R package MesKit package^6^ to each patient/lesion using the 30 COSMIC signatures (v2) as reference. After extraction, we calculated and compared the relative contribution of different signatures among different patients and lesions.

**Copy number analysis**

We applied Sequenza^7^ R package (version 3.0.0) to evaluate copy number (CN) states for each sample. Standard BAM files of samples with their matched normal controls were used as input to calculate the depth ratio considering both GC contents and data quantity. We further estimated the purity and ploidy of samples with the following parameters: breaks.method = ‘full’, gamma = 40, kmin = 5, gamma.pcf = 200, kmin.pcf = 200. Copy number gains and losses were defined as at least one copy more and one copy less than the estimated ploidy, respectively. Segments smaller than 500 kb were filtered and only autosomes were used in copy number analysis.

**Clonal status of somatic mutations**

Cancer cell fraction (CCF) of mutations were estimated by PyClone (v0.13.0) ^8^, which adjusted the variant allele frequencies (VAFs) of somatic mutations based on local copy numbers of the mutated loci and tumor purity. The merged CCF (${CCF}_{merged}$) of each mutation is computed by integrating multiple lesions as previously described ^9^.

${CCF}_{merged}=\left\{ \begin{aligned} \frac{\sum_{i=1}^{k} {CCF}_{i} \times d_{i}}{\sum_{i=1}^{k} d_{i}} & CCF<1 \\ 1 CCF\geq1 \end{aligned} \right.$

where $d_{i}$ and $\mathrm{CCF}_{i}$ refer to the sequencing depth and CCF estimation in lesion $i$, respectively. The clonal status of mutations are determined based on CCFs. A CCF value of 1 indicates that the mutation is present in 100% of the cancer cells in a sample, while a CCF value < 1 indicates that the mutation is present in a subset of the cancer cells in a sample and thus is subclonal. In each sample, a mutation is classified as clonal with upper bound of the 95% confidence interval (CI) of the CCF is $\geq1$; and otherwise subclonal ^10^. For tumors with multiple lesions, a mutation is considered subclonal when all of the following criteria are satisfied: (1) at least one region with upper bound of 95% CI of the CCF < 1; (2) at least one region with CCF < 0.5; (3) ${CCF}_{merged}$ of mutation m < 0.5 (the cut-off was chosen for its good performance in defining subclonalit­­y in simulated virtual tumors ^11, 12^.

**Sanger sequencing**

Genome DNA was isolated using the [TIANamp Genomic DNA Kit](https://en.tiangen.com/content/details_43_4224.html) (TIANGEN, DP304) according to the manufacturer’s recommendations. A NanoDrop One spectrophotometer (Thermo Scientific, Wilmington, DE) was used to assess DNA concentration. The average 260/280 ratio was 1.9, with a range of 1.8-2.09. Amplification primers were designed to produce one PCR amplicon for every single nucleotide (SNP) site (a total of 10 PCR primer pairs were designed). Amplification products were designed to cover the sequences and flanking the SNPs regions. Amplifications were performed using 50 ng of the extracted DNA in a 50 μl reaction with Q5® High-Fidelity 2X Master Mix on a BioRad T100TM Thermal Cycler System. After amplification, 2 µl of the PCR products were evaluated on a 2% agarose gel before Sanger sequencing.

**Detection of HBV integration sites**

Virus-Clip^13^ pipeline was used to identify the HBV integration sites based on WES data. First, it conducted reads alignment to HBV reference genomes (genotype A-H) using BWA-MEM. The HBV reference genomes were collected from HBVdb (https://hbvdb.lyon.inserm.fr/HBVdb/). Subsequently, the strain with the highest depth coverage of viral reads was selected as the reference strain for a given patient. Then, all soft-clipped reads were extracted and mapped to the human reference genome (hg19) by the BLASTN stand-alone version. The top match was reported as the junction of human and HBV sequence in a paired-end-assembled read, which is the HBV integration site. ANNOVAR was used to do the annotation for the integrated breakpoints.

**DNA methylation profiling**

FastQC v.0.11.9 was used for quality control. Then, TrimGalore v.0.6.5 was used to trim the Illumina adapter sequences and Bismark v.0.18.1 integrating bowtie2 v.2.2.3 was used to align the trimmed reads to the hg19 assembly of the human genome. The DNA methylation levels for individual CpG sites were quantified by counting the number of Cs versus the total number of reads for individual cytosines. Only CpG sites covered by at least 20 reads were retained to construct the multiregional methylation density profile for each patient.

**Phylogenetic tree and phyloepigenetic tree construction**

For each patient, we constructed the Euclidean distance matrix of somatic non-synonymous mutations and DNA methylation of all samples. The phylogenetic and phyloepigenetic trees were then inferred by the neighbor-joining algorithm in the ape package based on mutation and methylation matrix, respectively. Putative driver mutated genes and DDR genes were labeled on the trees according to their regional distributions. The phylogenetic trees and phyloepigenetic trees were visualized using MesKit package^6^. Pearson’s correlation coefficient was employed to calculate the similarity between the genetic and epigenetic distance matrices for each patient

**Immunohistochemistry and scoring**

mHCC tissue sections were formalin-fixed, paraffin-embedded, and H&E-stained in the light of the manufacturer’s instructions. Immunohistochemistry of 4-μm-sections was performed using Envision’s two steps protocol. Inflammatory cell surface markers were used to characterize the immune microenvironment including CD20 (Roche, 760-2531) for the B-lymphocytes, CD8 (ZSGB-BIO, ZM-0508) for the cytotoxic T-lymphocytes, CD4 (ZSGB-BIO, ZM-0418) for memory T-lymphocytes. We also characterized PD-1 (ZSGB-BIO, ZM-0381) and PD-L1 (Dako, 22C3). The secondary was an anti-rabbit/mouse IgG monoclonal antibody (DAKO Real Envision, Santa Clara, CA). To evaluate the densities of CD20^+^, CD4^+^, CD8^+^, and PD-1^+^ cells, tumor areas were screened by microscopy at low magnification (×100); at least three representative spot images were then selected and captured at ×200 magnification using a computerized system that include a Digital Slight DS-Fi1 camera installed on a Nikon Eclipse 80i light microscope (Nikon). Cells that were stained positive were automatically counted using inForm image analysis software (Perkin-Elmer Applied Biosystem; Hopkinton, MA, USA). Cell numbers were expressed as median per mm^2^. The specimens were evaluated independently to assess the expression of PD-L1 in tumor cells, T-cells and macrophages, CD20 in B-cells, as well as that of PD-1, CD4, and CD8 in T-cells ^14, 15^. Immunoreactivity for PD-L1 was scored using the combined positive score (CPS) method: the number of positive cells (including cancer cells, lymphocytes, and macrophages) / the total number of surviving tumor cells, multiplied by 100. The positive expression of PD-L1 was defined as a CPS ≥ 1.

**HLA typing**

HLA typing for HLA class-I genes (HLA-A, HLA-B and HLA-C) was carried out using OptiType^16^ and POLYSOLVER (v1.0)^17^ software for both tumor- and normal-bam files. To identify the most reliable patient-specific HLA-I type, we then integrated the results by OptiType and Polysolver of all samples from the same patient^18^.

**Neoantigen prediction**

Neoantigens were predicted from non-synonymous mutations (SNVs and INDELs) and HLA class-I gene type using the pVACseq toolkit^19^. All mutations were annotated by Variant Effect Predictor based on VCF files from Mutect2 and Stelka, and only those led to peptide changes were used for neoantigen prediction. The binding affinity between every mutant peptide and the respective patient’s HLA class-I molecules were predicted by NetMHC, NetMHCIIpan and NetMHCcons algorithms packed in pVACseq. Candidate neoantigens were identified as 8-11 mer peptides with a predicted binding strength of < 500 nM.

Discussion

In the present study, the clonal aspects of genetic and epigenetic intratumoral heterogeneity (ITH), the role of DDR alterations in evolution trajectory, as well as immune microenvironment profiles, and tumor response to immunotherapy of multifocal HCC (mHCC) harboring multifocal lesions were comprehensively investigated through multi-omics analysis. Substantial genetic and epigenetic ITH was identified in the evolution process of mHCC. Based on evolution trajectories derived from genetic and epigenetic aberrations, we underlined that DDR alterations, resulting from gene mutations and epigenetic modifications, fueled the early and late tumor evolution of mHCC. In addition, we also observed that DDR alterations within the patients were correlated with tumor immune microenvironment profiles, which exhibited good responses to the immunotherapy.

Our study offers the first insights into genetic and epigenetic clonal aspects of ITH in mHCC. For decades, previous investigations have tried to decipher the ITH of HCC mainly focused on patients with single tumors concerning genomes, proteomics, and transcriptomes, rather than mHCC harboring MLs, with epigenomes barely understood^6, 20-25^. Recent studies revealed that substantial evidence of spatial diversity was identified based on somatic mutation data in mHCCs^26, 27^. Similar to the results from these studies, only many trunk drivers (including *TP53*, and *AXIN1* mutations) were clonal in the present study, as would be expected of early events necessary for the establishment of the tumor. Subclonal evolution within tumors allows the reconstruction of the evolutionary history of phylogenetic relationships^28^. Some critical genetic alterations may appear to be clonal and misinterpreted through the conventional single-tumor-sampling approach. Thus, clonal diversity offers fertile soil for mHCC evolution, ultimately shaping genetic heterogeneity in multifocal lesions. The epigenetic mechanisms play an important role in ITH^29^, but the epigenetic heterogeneity has not been fully understood in mHCC. In the current study, we observed substantial epigenetic ITH within the individual tumor as well as between tumors, typically involving thousands of loci in a particular genome. Although these aberrant methylation alterations are present in the monoclonal origin from a common ancestor or independently arose in distinct tumor subpopulations, the epigenome continues to evolve in parallel to the acquisition of driving and passenger copy-number mutations^30^. Also, we found that the global methylation levels of tumor thrombi in HCC1, HCC5 and HCC7 were lower than those of the paired primary tumors, indicating evident advanced subclonal complexity evolution. This observation conforms to the emerging view that high epigenetic ITH is correlated with the progression of tumor patients^31^. Furthermore, tumor suppressors (*PTCH1, RASSF2,* and *GSTP1*) and oncogenes (*KEAP1*) were variously methylated at their CpG island (CGI)-promoters, suggesting that these genes might be [diverse](#en/zh/diverse)ly reprogrammed in different sub-populations. Interestingly, *KEAP1* was both mutated and methylated at the CGI-promoter. The interaction between genetic and epigenetic variations of *KEAP1* needs to be explored and more evidence is needed to support the possibility of such an interaction. More importantly, phyloepigenetic trees construction strongly emphasizes the importance of epigenetic ITH in the evolution course of mHCC. Notably, the topologies of phylogenetic trees and phyloepigenetic trees revealed high concordance in all cases except HCC7, indicating the methylation participation (either collaborative or independent of the genome) in subclonal diversification. The integrated phylogenetic and phyloepigenetic landscapes highlight new insight into a co-evolutionary association between the genetic and epigenetic heterogeneity in mHCCs with MLs. Consistent with our reports, the genetic and epigenetic co-evolutionary has been shown in various human cancers, such as prostate and esophageal cancers^32, 33^. The interplay of tumor heterogeneities at the genetic and epigenetic levels and their molecular trajectories are vital to understanding the behavior and natural history of mHCC.

DNA damage repair (DDR) system is formed to maintain genome stability which is a driving force promoting continuous modification of tumor genomes and leading to clonal evolution^34^. DDR alterations, resulting from DDR gene mutations or epigenetic modifications, have been involved in carcinogenesis and immunotherapy response^35^. Recently, evidence indicates that mutations in DDR genes were identified in patients with HCC^36^. To the best of our knowledge, our current study was the first to report that DDR alterations observed in mHCC are linked to tumor immune microenvironment and tumor progression in both genome and epigenome evolution of MLs, allowing for investigation of concordant tumor evolutionary trajectories as inferred by both their DNA mutations and methylation. Intriguingly, all patients except HCC5 had DDR-associated signatures including Signature 3, 6, 14, 15, 20, and 26. Notably, mutations in *TP53* were identified in all seven patients, predominantly enriched on the trunks of six patients. Restoration of *p53* function in these patients could trigger apoptosis of tumor cells that have extensive DNA damage^37^. Frequent *TP53* mutations as truncal events in our study highlight the importance of this gene as a founder in evolution. The abrogation of *BRCA1* or *BRCA2,* which are cancer suppressors, causes DSB accumulation and induces anti-cancer immunity^38, 39^. We identified poly (ADP-ribose) polymerase inhibitors (PARPi) targeted *BRCA2* mutations as branch events in four patients. These findings suggest that DDR alterations resulting from DDR gene mutations not only function as an anticancer barrier in early-stage tumorigenesis, but also participate in later-stage tumors. At the epigenetic level, we also observed aberrant methylation of DDR genes in different MLs of all patients. Notably, most of these methylation alterations including *MLH3*, *RNF8* and *PTCH* were trunk events, which indicates that dysregulated DNA methylation of DDR genes is an early event parallel to or even prior to genetic alterations in mHCC evolution. Based on WES and WGBS data, we undefined that DDR alterations were linked to both genetic and epigenetic tumor evolution in mHCC.

Endogenous causes of DNA damage within the evolving tumor and extrinsic factors from the tumor microenvironment such as immune cells (immunoediting of the tumor) have been proved to be potential drivers of tumor evolution^34, 40^. DDR alteration is one leading cause of genetic instability, contributing to activating immune signaling and thus serving as a potential predictive biomarker for immunotherapy^35, 41^. In the current study, among four patients who received anti-PD-1 immunotherapy, three responders, including PR and SD patients, had DDR-associated signatures, with increased tumor neoantigen burden (TNB) and increased tumor mutational burden (TMB). More generally, the correlation between increased TNB, increased TMB and better response to immunotherapy has been retrospectively confirmed in patients with non-small-cell lung cancer, melanoma, urothelial bladder carcinoma, and head-and-neck squamous cell carcinoma^32, 33^. Besides, the responders tended to have higher densities of infiltrating CD8^+^ T lymphocytes and PD-L1 expression, compared to non-responder (PD). Various DDR alterations have been linked to the modulation of immune checkpoint expression in tumor cell^35^. Our findings underscore the potentially important role of DDR alterations in driving sensitivity and response to immunotherapy. However, in the present study, the sample size is insufficient to perform the statistical test, and the results are limited. It warrants further study to manifest how the above-mentioned genetic and epigenetic ITH affects the development of DDR targets and the efficacy of immunotherapy.

In summary, the present work provided a comprehensive evaluation of the genetic and epigenetic ITH of mHCC and found a novel understanding of the importance of DDR alterations resulting from DDR gene mutations or epigenetic modifications that might be implicated in tumor evolution. Of the patients receiving immunotherapy, DDR alterations are noteworthy, as they suggest a potential for their use as a novel predictor for therapeutic efficacy.

Figure. S1.


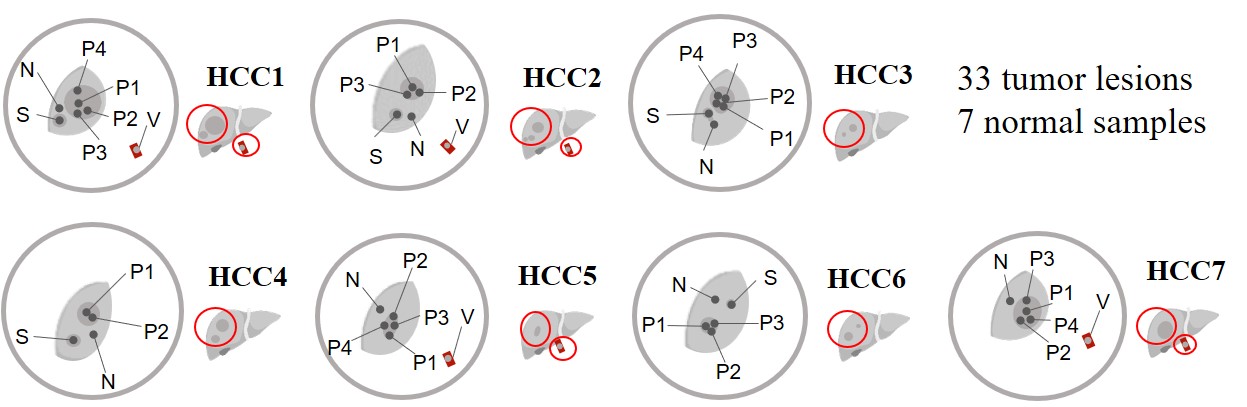


**Figure. S1.** **Schematic diagram of sampling in seven mHCC patients.**

Figure. S2.


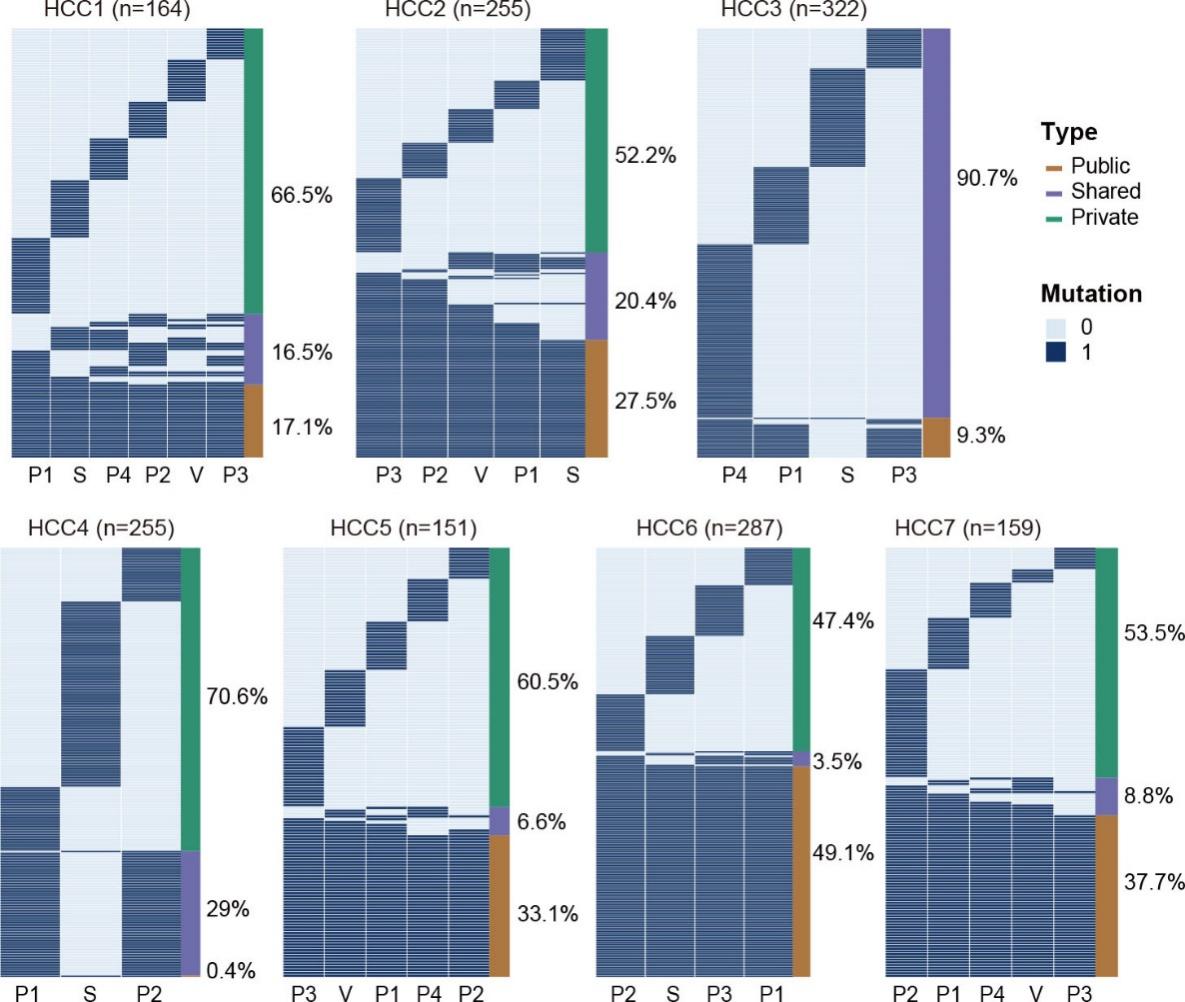


**Figure. S2. Mutational profiles of seven mHCC patients.** Grid colors indicate the presence (blue) or absence (gray) of somatic nonsynonymous mutations in samples from the same patient. Mutations were grouped into three classes based on their regional distribution: public mutations (brown bar), shared mutations (purple bar), and private mutations (green bar). The proportion of each classification is indicated in the legend.

Figure. S3.


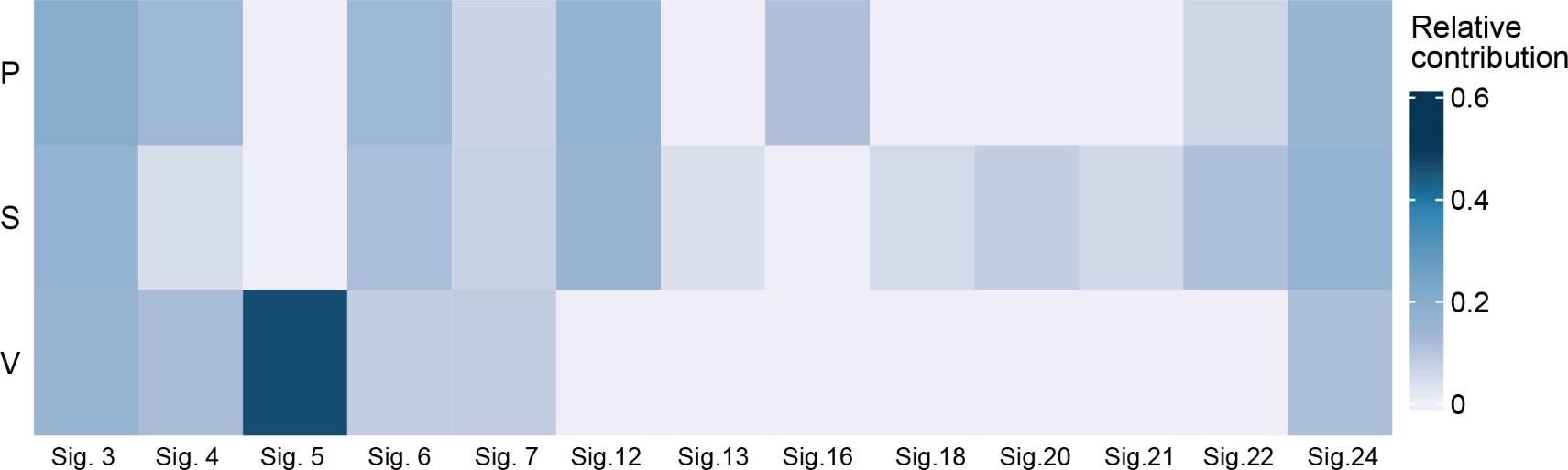


**Figure. S3. The relative contribution of each COSMIC v2 signature for different tumor lesions across seven mHCC patients.** P: primary tumor; S: Satellite nodule; V: Portal vein tumor thrombus. Only signatures with relative contribution > 0.1 were retained.

Figure. S4.

**
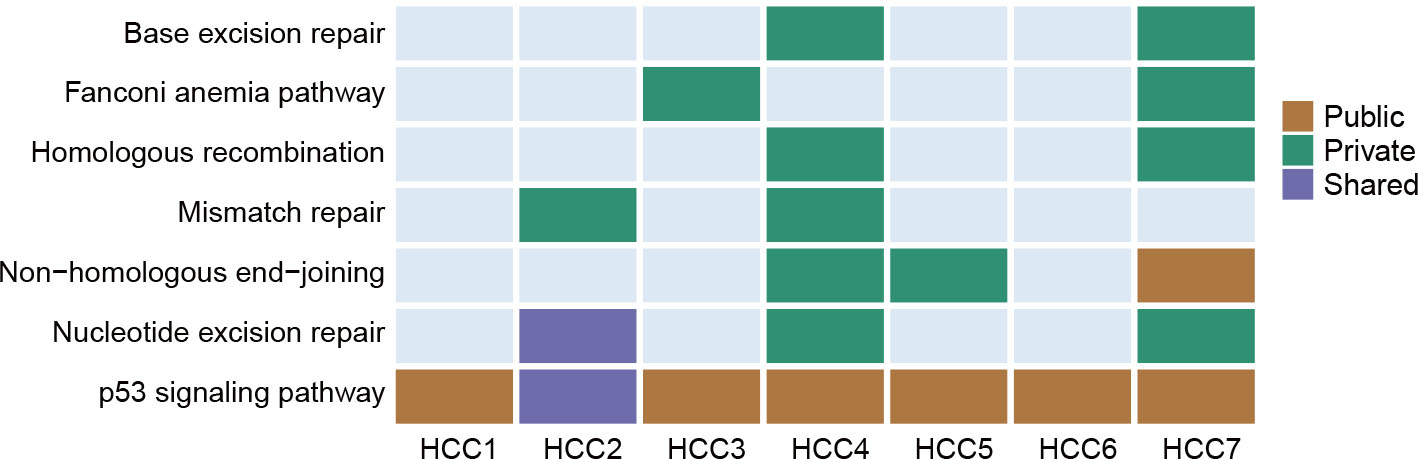
**

**Figure. S4. DDR pathways with genetic mutations were identified for each mHCC patient.** Green grid indicates that the corresponding DDR pathway was mutated.

Figure. S5.


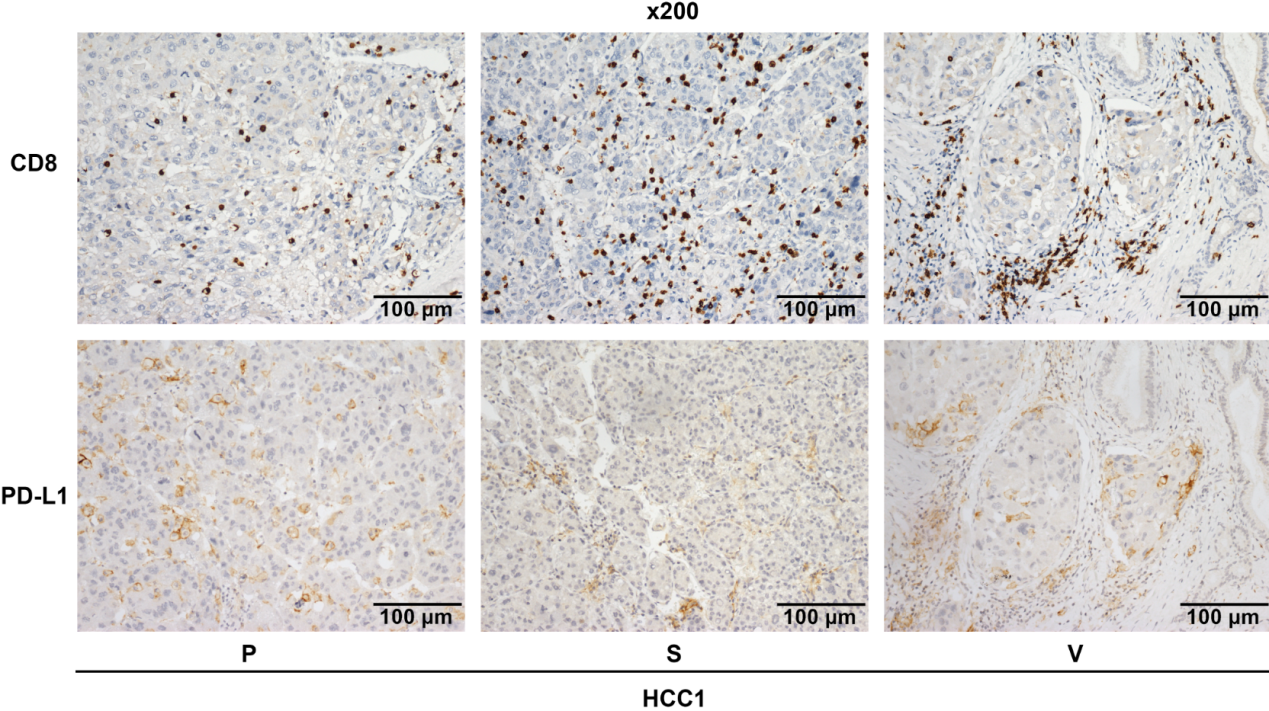


Figure. S5. Expression of CD8 and PD-L1 in tumor lesions of HCC1 detected by immunohistochemistry.

Figure. S6.


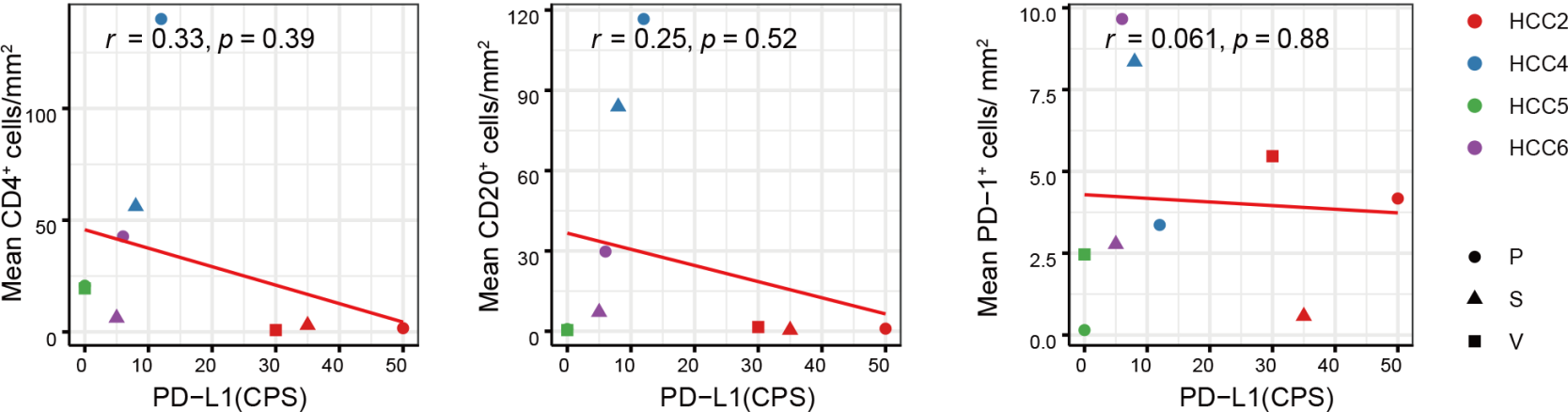


Figure. S6. Pearson’s correlation analysis was performed between CD4^+^T cell densities, CD20^+^B cell densities, PD-1^+^ lymphocytes densities and expressions of PD-L1 (CPS score).

Table S1. Clinicopathological parameters of seven mHCC patients.

| Patient | Age | Gender | AFP (ng/L) | TNM stage | Samples | HBsAg | HBV (IU/ml) | Alcohol | Smoking | Recurrence/ Metastasis | Immunotherapy/Response | Status |
| --- | --- | --- | --- | --- | --- | --- | --- | --- | --- | --- | --- | --- |
| HCC1 | 44 | M | 53798 | T4N0M0 | P (4), S (1), V (1) | + | 6.12*10^6 | - | + | Early recurrence | - | Dead |
| HCC2 | 39 | M | 38564 | T3bN0M0 | P (3), S (1), V (1) | + | 59.4 | - | + | Lung metastasis | Toripalimab/PR | Alive |
| HCC3 | 39 | M | 130.4 | T2N0M0 | P (4), S (1) | + | 5.98*10^4 | - | - | Late recurrence | - | Alive |
| HCC4 | 68 | M | 9771 | T1bN0M0 | P (2), S (1) | - | 0 | + | + | - | Toripalimab/SD | Alive |
| HCC5 | 48 | M | 6.77 | T3bN0M0 | P (4), V (1) | + | 4.07*10^5 | + | + | Early recurrence | Toripalimab/PD | Dead |
| HCC6 | 54 | M | 160.2 | T2N0M0 | P (3), S (1) | + | 1.26*10^5 | - | - | - | Toripalimab/PR | Alive |
| HCC7 | 55 | M | 17852 | T3bN0M0 | P (4), V (1) | + | 28.1 | - | - | Multiple metastases | - | Dead |

AFP, A-fetoprotein; HBsAg, Hepatitis B surface antigen. HBV DNA copy number was measured by qPCR assay. Assessments for therapeutic response were according to Response Evaluation Criteria in Solid Tumors, RECIST, version 1.

**Table S2. HBV integration sites identified by WES.**

| **Sample** | **HBV genotype** | **Genomic position** | **Read count** | **Gene region** | **Gene symbol** | **Cytoband** | **Integration event** |
| --- | --- | --- | --- | --- | --- | --- | --- |
| HCC1-P1 | B | chr12:70133608 | 2 | UTR5 | RAB3IP | 12q15 | HCC1-E1 |
| HCC1-P2 | B | chr7:49956690 | 1 | downstream | VWC2 | 7p12.2 | HCC1-E10 |
| HCC1-V | B | chr7:49956690 | 1 | downstream | VWC2 | 7p12.2 | HCC1-E10 |
| HCC1-P1 | B | chr8:105392052 | 27 | UTR3 | DPYS | 8q22.3 | HCC1-E11 |
| HCC1-P2 | B | chr8:105392052 | 1 | UTR3 | DPYS | 8q22.3 | HCC1-E11 |
| HCC1-P2 | B | chr8:105392052 | 55 | UTR3 | DPYS | 8q22.3 | HCC1-E11 |
| HCC1-P3 | B | chr8:105392052 | 1 | UTR3 | DPYS | 8q22.3 | HCC1-E11 |
| HCC1-P3 | B | chr8:105392052 | 2 | UTR3 | DPYS | 8q22.3 | HCC1-E11 |
| HCC1-P3 | B | chr8:105392052 | 54 | UTR3 | DPYS | 8q22.3 | HCC1-E11 |
| HCC1-P4 | B | chr8:105392052 | 56 | UTR3 | DPYS | 8q22.3 | HCC1-E11 |
| HCC1-S | B | chr8:105392052 | 8 | UTR3 | DPYS | 8q22.3 | HCC1-E11 |
| HCC1-N | B | chr8:104340562 | 6 | exonic | FZD6 | 8q22.3 | HCC1-E12 |
| HCC1-P1 | B | chr14:73577791 | 2 | exonic | RBM25 | 14q24.2 | HCC1-E2 |
| HCC1-P4 | B | chr17:40275419 | 2 | downstream | HSPB9 | 17q21.2 | HCC1-E3 |
| HCC1-N | B | chr17:72875481 | 2 | intronic | FADS6 | 17q25.1 | HCC1-E4 |
| HCC1-P3 | B | chr19:27732020 | 2 | intergenic | NONE-LINC00662 | 19q11 | HCC1-E5 |
| HCC1-P3 | B | chr1:91676157 | 2 | intergenic | ZNF644-HFM1 | 1p22.2 | HCC1-E6 |
| HCC1-V | B | chr1:91676157 | 1 | intergenic | ZNF644-HFM1 | 1p22.2 | HCC1-E6 |
| HCC1-V | B | chr1:91675876 | 2 | intergenic | ZNF644-HFM1 | 1p22.2 | HCC1-E6 |
| HCC1-P1 | B | chr2:207175086 | 2 | exonic | ZDBF2 | 2q33.3 | HCC1-E7 |
| HCC1-P3 | B | chr2:243153910 | 2 | intergenic | LOC728323-NONE | 2q37.3 | HCC1-E8 |
| HCC1-N | B | chr6:31687807 | 2 | intronic | LY6G6C | 6p21.33 | HCC1-E9 |
| HCC3-P1 | C | chr12:105815748 | 4 | intergenic | C12orf75-CASC18 | 12q23.3 | HCC3-E1 |
| HCC3-P1 | C | chr22:26800064 | 6 | intergenic | SEZ6L-ASPHD2 | 22q12.1 | HCC3-E10 |
| HCC3-P3 | C | chr2:207007545 | 2 | exonic | NDUFS1 | 2q33.3 | HCC3-E11 |
| HCC3-P1 | C | chr4:17584079 | 2 | intronic | LAP3 | 4p15.32 | HCC3-E12 |
| HCC3-P2 | C | chr5:1295704 | 1 | upstream | TERT | 5p15.33 | HCC3-E13 |
| HCC3-P2 | C | chr5:1295704 | 3 | upstream | TERT | 5p15.33 | HCC3-E13 |
| HCC3-N | C | chr5:175764311 | 7 | intronic | SIMC1 | 5q35.2 | HCC3-E14 |
| HCC3-N | C | chr5:175764078 | 1 | exonic | SIMC1 | 5q35.2 | HCC3-E14 |
| HCC3-P1 | C | chr9:118555530 | 1 | intergenic | LOC101928775-LINC00474 | 9q33.1 | HCC3-E15 |
| HCC3-P1 | C | chr9:118555559 | 1 | intergenic | LOC101928775-LINC00474 | 9q33.1 | HCC3-E15 |
| HCC3-P1 | C | chr12:123237407 | 6 | UTR5 | DENR | 12q24.31 | HCC3-E2 |
| HCC3-P3 | C | chr12:123237399 | 1 | UTR5 | DENR | 12q24.31 | HCC3-E2 |
| HCC3-P4 | C | chr12:123237412 | 4 | UTR5 | DENR | 12q24.31 | HCC3-E2 |
| HCC3-P1 | C | chr14:107013374 | 6 | intergenic | LINC00221-MIR7641-2 | 14q32.33 | HCC3-E3 |
| HCC3-P4 | C | chr17:12564255 | 2 | upstream | MYOCD | 17p12 | HCC3-E4 |
| HCC3-P1 | C | chr17:4579381 | 4 | exonic | PELP1 | 17p13.2 | HCC3-E5 |
| HCC3-P1 | C | chr19:36213905 | 8 | exonic | KMT2B | 19q13.12 | HCC3-E6 |
| HCC3-P1 | C | chr19:36213660 | 7 | intronic | KMT2B | 19q13.12 | HCC3-E6 |
| HCC3-P3 | C | chr19:36213899 | 4 | exonic | KMT2B | 19q13.12 | HCC3-E6 |
| HCC3-P3 | C | chr19:36213660 | 2 | intronic | KMT2B | 19q13.12 | HCC3-E6 |
| HCC3-P4 | C | chr19:36213839 | 3 | intronic | KMT2B | 19q13.12 | HCC3-E6 |
| HCC3-P1 | C | chr19:54458012 | 2 | upstream | CACNG8 | 19q13.42 | HCC3-E7 |
| HCC3-P1 | C | chr1:154042956 | 2 | intronic | NUP210L | 1q21.3 | HCC3-E8 |
| HCC3-P1 | C | chr20:9674174 | 2 | intronic | PAK7 | 20p12.2 | HCC3-E9 |
| HCC5-P1 | C | chr11:597520 | 4 | exonic | PHRF1 | 11p15.5 | HCC5-E1 |
| HCC5-P3 | C | chr13:40119560 | 8 | intronic | LHFP | 13q14.11 | HCC5-E2 |
| HCC5-P3 | C | chr15:77212927 | 2 | intergenic | SCAPER-RCN2 | 15q24.3 | HCC5-E3 |
| HCC5-P1 | C | chr4:31813525 | 3 | intergenic | LOC102723778-LOC102723828 | 4p15.1 | HCC5-E4 |
| HCC5-P4 | C | chr4:31813493 | 1 | intergenic | LOC102723778-LOC102723828 | 4p15.1 | HCC5-E4 |
| HCC5-P3 | C | chr5:140515535 | 2 | exonic | PCDHB5 | 5q31.3 | HCC5-E5 |
| HCC5-P2 | C | chr7:29356355 | 2 | intronic | CHN2 | 7p14.3 | HCC5-E6 |
| HCC5-P3 | C | chr8:1871110 | 3 | intronic | ARHGEF10 | 8p23.3 | HCC5-E7 |
| HCC5-P1 | C | chr9:21979500 | 9 | intronic | CDKN2A | 9p21.3 | HCC5-E8 |
| HCC5-P1 | C | chr9:22030707 | 6 | intronic | CDKN2B-AS1 | 9p21.3 | HCC5-E8 |
| HCC5-P2 | C | chr9:21979500 | 3 | intronic | CDKN2A | 9p21.3 | HCC5-E8 |
| HCC5-P4 | C | chr9:21979500 | 4 | intronic | CDKN2A | 9p21.3 | HCC5-E8 |
| HCC5-V | C | chr9:21979500 | 4 | intronic | CDKN2A | 9p21.3 | HCC5-E8 |
| HCC5-P3 | C | chrX:46857455 | 6 | intronic | JADE3 | Xp11.23 | HCC5-E9 |
| HCC6-P1 | C | chr19:36212728 | 17 | intronic | KMT2B | 19q13.12 | HCC6-E1 |
| HCC6-P1 | C | chr19:36212717 | 33 | intronic | KMT2B | 19q13.12 | HCC6-E1 |
| HCC6-P2 | C | chr19:36212728 | 20 | intronic | KMT2B | 19q13.12 | HCC6-E1 |
| HCC6-P2 | C | chr19:36212718 | 45 | intronic | KMT2B | 19q13.12 | HCC6-E1 |
| HCC6-P2 | C | chr19:36212721 | 1 | intronic | KMT2B | 19q13.12 | HCC6-E1 |
| HCC6-P3 | C | chr19:36212728 | 11 | intronic | KMT2B | 19q13.12 | HCC6-E1 |
| HCC6-P3 | C | chr19:36212727 | 30 | intronic | KMT2B | 19q13.12 | HCC6-E1 |
| HCC6-P3 | C | chr19:36212730 | 2 | intronic | KMT2B | 19q13.12 | HCC6-E1 |
| HCC6-S | C | chr19:36212728 | 15 | intronic | KMT2B | 19q13.12 | HCC6-E1 |
| HCC6-S | C | chr19:36212717 | 47 | intronic | KMT2B | 19q13.12 | HCC6-E1 |
| HCC6-S | C | chr19:36212717 | 1 | intronic | KMT2B | 19q13.12 | HCC6-E1 |
| HCC6-S | C | chr19:36212721 | 1 | intronic | KMT2B | 19q13.12 | HCC6-E1 |
| HCC6-P1 | C | chr22:50356455 | 49 | exonic | PIM3 | 22q13.33 | HCC6-E2 |
| HCC6-P1 | C | chr22:50356482 | 3 | exonic | PIM3 | 22q13.33 | HCC6-E2 |
| HCC6-P2 | C | chr22:50356455 | 38 | exonic | PIM3 | 22q13.33 | HCC6-E2 |
| HCC6-P2 | C | chr22:50356482 | 5 | exonic | PIM3 | 22q13.33 | HCC6-E2 |
| HCC6-P3 | C | chr22:50356455 | 2 | exonic | PIM3 | 22q13.33 | HCC6-E2 |
| HCC6-P3 | C | chr22:50356455 | 63 | exonic | PIM3 | 22q13.33 | HCC6-E2 |
| HCC6-P3 | C | chr22:50356482 | 6 | exonic | PIM3 | 22q13.33 | HCC6-E2 |
| HCC6-S | C | chr22:50356455 | 65 | exonic | PIM3 | 22q13.33 | HCC6-E2 |
| HCC6-S | C | chr22:50356482 | 7 | exonic | PIM3 | 22q13.33 | HCC6-E2 |
| HCC7-P4 | B | chr13:107717459 | 2 | intergenic | LINC00443-FAM155A | 13q33.3 | HCC7-E1 |
| HCC7-P2 | B | chr15:76578834 | 4 | intronic | ETFA | 15q24.2 | HCC7-E2 |
| HCC7-P4 | B | chr8:48272595 | 1 | intronic | SPIDR | 8q11.21 | HCC7-E3 |
| HCC7-P4 | B | chr8:48272594 | 8 | intronic | SPIDR | 8q11.21 | HCC7-E3 |
| HCC7-P1 | B | chr8:146169226 | 2 | intronic | ZNF16 | 8q24.3 | HCC7-E4 |

Table S3. Discrimination of IM/MO in mHCC based on WES

| **Patient** | **Secondary lesions** | **Shared mutations (%)** | **Shared CNAs (%)** | **Shared HBV integration site** | **Phylogeny** | **IM/MO diagnosis** |
| --- | --- | --- | --- | --- | --- | --- |
| HCC1 | S, V | 48.45 (S) | 63.84 (S) | Yes | Close | IM |
|  |  | 61.81 (V) | 56.12 (V) |  |  | IM |
| HCC2 | S, V | 60.82 (S) | 36.27 (S) | No | Close | IM |
|  |  | 67.21 (V) | 45.44 (V) |  |  | IM |
| HCC3 | S | 2.69 | 18.75 | No | Distant | MO |
| HCC4 | S | 0.54 | 4.48 | No | Distant | MO |
| HCC5 | V | 69.54 | 22.36 | Yes | Close | IM |
| HCC6 | S | 76.22 | 77.84 | Yes | Close | IM |
| HCC7 | V | 86.08 | 71.22 | No | Close | IM |

**REFERENCES**

1. Li, H. & Durbin, R. Fast and accurate short read alignment with Burrows-Wheeler transform. *Bioinformatics* **25**, 1754-1760 (2009).

2. Cibulskis, K. *et al.* Sensitive detection of somatic point mutations in impure and heterogeneous cancer samples. *Nat Biotechnol* **31**, 213-219 (2013).

3. Saunders, C.T. *et al.* Strelka: accurate somatic small-variant calling from sequenced tumor-normal sample pairs. *Bioinformatics* **28**, 1811-1817 (2012).

4. Chen, X. *et al.* Manta: rapid detection of structural variants and indels for germline and cancer sequencing applications. *Bioinformatics* **32**, 1220-1222 (2016).

5. Wang, K., Li, M. & Hakonarson, H. ANNOVAR: functional annotation of genetic variants from high-throughput sequencing data. *Nucleic Acids Res* **38**, e164 (2010).

6. Liu, M. *et al.* MesKit: a tool kit for dissecting cancer evolution of multi-region tumor biopsies through somatic alterations. *Gigascience* **10** (2021).

7. Favero, F. *et al.* Sequenza: allele-specific copy number and mutation profiles from tumor sequencing data. *Ann Oncol* **26**, 64-70 (2015).

8. Roth, A. *et al.* PyClone: statistical inference of clonal population structure in cancer. *Nat Methods* **11**, 396-398 (2014).

9. Hu, Z. *et al.* Quantitative evidence for early metastatic seeding in colorectal cancer. *Nat Genet* **51**, 1113-1122 (2019).

10. McGranahan, N. *et al.* Clonal status of actionable driver events and the timing of mutational processes in cancer evolution. *Sci Transl Med* **7**, 283ra254 (2015).

11. Sun, R. *et al.* Between-region genetic divergence reflects the mode and tempo of tumor evolution. *Nat Genet* **49**, 1015-1024 (2017).

12. Caswell-Jin, J.L. *et al.* Clonal replacement and heterogeneity in breast tumors treated with neoadjuvant HER2-targeted therapy. *Nat Commun* **10**, 657 (2019).

13. Ho, D.W., Sze, K.M. & Ng, I.O. Virus-Clip: a fast and memory-efficient viral integration site detection tool at single-base resolution with annotation capability. *Oncotarget* **6**, 20959-20963 (2015).

14. Ye, Y. *et al.* Interaction of B7-H1 on intrahepatic cholangiocarcinoma cells with PD-1 on tumor-infiltrating T cells as a mechanism of immune evasion. *J Surg Oncol* **100**, 500-504 (2009).

15. Muenst, S. *et al.* The presence of programmed death 1 (PD-1)-positive tumor-infiltrating lymphocytes is associated with poor prognosis in human breast cancer. *Breast Cancer Research and Treatment* **139**, 667-676 (2013).

16. Szolek, A. *et al.* OptiType: precision HLA typing from next-generation sequencing data. *Bioinformatics* **30**, 3310-3316 (2014).

17. Shukla, S.A. *et al.* Comprehensive analysis of cancer-associated somatic mutations in class I HLA genes. *Nat Biotechnol* **33**, 1152-1158 (2015).

18. Yi, J., Chen, L., Xiao, Y., Zhao, Z. & Su, X. Investigations of sequencing data and sample type on HLA class Ia typing with different computational tools. *Brief Bioinform* **22** (2021).

19. Hundal, J. *et al.* pVACtools: A Computational Toolkit to Identify and Visualize Cancer Neoantigens. *Cancer Immunol Res* **8**, 409-420 (2020).

20. Zhang, C., El-Kebir, M. & Ochoa, I. Moss enables high sensitivity single-nucleotide variant calling from multiple bulk DNA tumor samples. *Nature Communications* **12** (2021).

21. Losic, B. *et al.* Intratumoral heterogeneity and clonal evolution in liver cancer. *Nat Commun* **11**, 291 (2020).

22. Furuta, M. *et al.* Whole genome sequencing discriminates hepatocellular carcinoma with intrahepatic metastasis from multi-centric tumors. *J Hepatol* **66**, 363-373 (2017).

23. Xue, R. *et al.* Variable Intra-Tumor Genomic Heterogeneity of Multiple Lesions in Patients With Hepatocellular Carcinoma. *Gastroenterology* **150**, 998-1008 (2016).

24. Dong, X. *et al.* Single-cell analysis reveals the intra-tumor heterogeneity and identifies MLXIPL as a biomarker in the cellular trajectory of hepatocellular carcinoma. *Cell Death Discov* **7**, 14 (2021).

25. Takeda, H. *et al.* Multiregional whole-genome sequencing of hepatocellular carcinoma with nodule-in-nodule appearance reveals stepwise cancer evolution. *J Pathol* **252**, 398-410 (2020).

26. Lin, D.C. *et al.* Genomic and Epigenomic Heterogeneity of Hepatocellular Carcinoma. *Cancer Res* **77**, 2255-2265 (2017).

27. Baylin, S.B. & Jones, P.A. A decade of exploring the cancer epigenome — biological and translational implications. *Nature Reviews Cancer* **11**, 726-734 (2011).

28. Campbell, P.J. *et al.* Subclonal phylogenetic structures in cancer revealed by ultra-deep sequencing. *Proc Natl Acad Sci U S A* **105**, 13081-13086 (2008).

29. Vitale, I., Shema, E., Loi, S. & Galluzzi, L. Intratumoral heterogeneity in cancer progression and response to immunotherapy. *Nature Medicine* **27**, 212-224 (2021).

30. Hama, N. *et al.* Epigenetic landscape influences the liver cancer genome architecture. *Nat Commun* **9**, 1643 (2018).

31. Mazor, T., Pankov, A., Song, J.S. & Costello, J.F. Intratumoral Heterogeneity of the Epigenome. *Cancer Cell* **29**, 440-451 (2016).

32. Brocks, D. *et al.* Intratumor DNA methylation heterogeneity reflects clonal evolution in aggressive prostate cancer. *Cell Rep* **8**, 798-806 (2014).

33. Hao, J.J. *et al.* Spatial intratumoral heterogeneity and temporal clonal evolution in esophageal squamous cell carcinoma. *Nat Genet* **48**, 1500-1507 (2016).

34. Pilie, P.G., Tang, C., Mills, G.B. & Yap, T.A. State-of-the-art strategies for targeting the DNA damage response in cancer. *Nat Rev Clin Oncol* **16**, 81-104 (2019).

35. Chabanon, R.M. *et al.* Targeting the DNA damage response in immuno-oncology: developments and opportunities. *Nat Rev Cancer* **21**, 701-717 (2021).

36. Lin, J. *et al.* Alterations in DNA Damage Repair Genes in Primary Liver Cancer. *Clin Cancer Res* **25**, 4701-4711 (2019).

37. Pearl, L.H., Schierz, A.C., Ward, S.E., Al-Lazikani, B. & Pearl, F.M. Therapeutic opportunities within the DNA damage response. *Nat Rev Cancer* **15**, 166-180 (2015).

38. Mackenzie, K.J. *et al.* cGAS surveillance of micronuclei links genome instability to innate immunity. *Nature* **548**, 461-465 (2017).

39. Tarsounas, M. & Sung, P. The antitumorigenic roles of BRCA1–BARD1 in DNA repair and replication. *Nature Reviews Molecular Cell Biology* **21**, 284-299 (2020).

40. Craig, A.J., von Felden, J., Garcia-Lezana, T., Sarcognato, S. & Villanueva, A. Tumour evolution in hepatocellular carcinoma. *Nat Rev Gastroenterol Hepatol* **17**, 139-152 (2020).

41. Mouw, K.W., Goldberg, M.S., Konstantinopoulos, P.A. & D'Andrea, A.D. DNA Damage and Repair Biomarkers of Immunotherapy Response. *Cancer Discov* **7**, 675-693 (2017).
